# tiRNA-Val-CAC-2 interacts with FUBP1 to promote pancreatic cancer metastasis by activating *c‑MYC* transcription

**DOI:** 10.1038/s41388-024-02991-9

**Published:** 2024-03-05

**Authors:** Qunli Xiong, Yaguang Zhang, Yongfeng Xu, Yang Yang, Zhiwei Zhang, Ying Zhou, Su Zhang, Lian Zhou, Xiaowen Wan, Xiaojuan Yang, Zhu Zeng, Jinlu Liu, Ying Zheng, Junhong Han, Qing Zhu

**Affiliations:** 1 Division of Abdominal Tumor Multimodality Treatment, Cancer Center, Department of General Surgery, West China Hospital, Sichuan University, Chengdu, 610041 China; 2 Department of Biotherapy, Cancer Center and State Laboratory of Biotherapy, and Frontiers Science Center for Disease-related Molecular Network, West China Hospital, Sichuan University, Chengdu, 610041 China

**Keywords:** Metastasis, Pancreatic cancer

## Abstract

Cumulative studies have established the significance of transfer RNA-derived small RNA (tsRNA) in tumorigenesis and progression. Nevertheless, its function and mechanism in pancreatic cancer metastasis remain largely unclear. Here, we screened and identified tiRNA-Val-CAC-2 as highly expressed in pancreatic cancer metastasis samples by tsRNA sequencing. We also observed elevated levels of tiRNA-Val-CAC-2 in the serum of pancreatic cancer patients who developed metastasis, and patients with high levels of tiRNA-Val-CAC-2 exhibited a worse prognosis. Additionally, knockdown of tiRNA-Val-CAC-2 inhibited the metastasis of pancreatic cancer in vivo and in vitro, while overexpression of tiRNA-Val-CAC-2 promoted the metastasis of pancreatic cancer. Mechanically, we discovered that tiRNA-Val-CAC-2 interacts with FUBP1, leading to enhanced stability of FUBP1 protein and increased FUBP1 enrichment in the *c-MYC* promoter region, thereby boosting the transcription of *c-MYC*. Of note, rescue experiments confirmed that tiRNA-Val-CAC-2 could influence pancreatic cancer metastasis *via* FUBP1-mediated *c-MYC* transcription. These findings highlight a potential novel mechanism underlying pancreatic cancer metastasis, and suggest that both tiRNA-Val-CAC-2 and FUBP1 could serve as promising prognostic biomarkers and potential therapeutic targets for pancreatic cancer.

## Introduction

Pancreatic cancer has become a prominent public health problem with high morbidity and mortality. According to the global cancer data statistics in 2020, the morbidity and mortality rate of pancreatic cancer ranked 14th and 7th, respectively [1], and it is estimated to become the second leading cause of cancer death by 2030 [2]. Pancreatic cancer patients commonly exhibit inadequate specific clinical symptoms during the initial stages and are thus often diagnosed at advanced stages, with high propensities for metastasis. In particular, the liver frequently serves as the primary destination for pancreatic cancer metastasis, leading to a median survival period below 12 months, thereby posing critical threats to human health [3]. Therefore, screening and identifying early diagnostic markers or potential therapeutic targets for pancreatic cancer has significant implications for improving the quality of life and prognosis of patients with this disease.

Non-coding RNA (ncRNA) refers to a class of RNA which is unable to encode proteins. Recently, the research on the regulatory function of ncRNA in tumors and its interactive relationship with tumor occurrence and development has become a hot topic in the biomedical field [4–6]. tRNA-derived small RNA (tsRNA) is a small fragment RNA of specific size ranging from 15–40 nt generated by specialized nucleases (such as Dicer and angiogenin) cleaving tRNA loops in specific cells or tissues [7, 8]. Accumulated evidence shows that tsRNAs have extensive biological functions [9, 10], including participation in cell and tissue stress responses [11], protein translation regulation [10], stem cell biology [12], ribosome biogenesis [13], transposon regulation [14], etc. For example, Mo et al. identified low expression of 5′-tiRNA-Val in breast cancer tissue and found negative correlation between the content of 5′-tiRNA-Val in patient serum and tumor stage as well as lymph node metastasis. Further research revealed that 5′-tiRNA-Val inhibits the FZD3/Wnt/β-catenin signaling pathway and becomes a new tumor suppressor, which may become a potential diagnostic biomarker for breast cancer [15]. Han et al. discovered significant increase of 33 nt tiRNA-Gly in papillary thyroid cancer based on tRFs and tiRNA sequencing. Mechanistic studies showed that tiRNA-Gly directly binds to the UHM domain of splicing related RNA-binding protein RBM17 [16]. Tao and colleagues found the distribution of tsRNAs in human colorectal cancer tissue and confirmed that 5’tiRNA-His-GTG is upregulated in colorectal cancer tissue. In vivo and in vitro experiments revealed the carcinogenic effect of 5’tiRNA-His-GTG in colorectal cancer and found that targeting 5′tiRNA-His-GTG can induce cancer cell apoptosis [17]. Jin et al. identified pancreatic cancer-related tsRNAs using RNA sequencing, RT-qPCR and in situ hybridization (ISH) in human serum, tissue, cancer cells, and mouse models. Changes in tsRNA profiles in serum and tumor tissue provide new biomarkers for early diagnosis and prognosis of pancreatic cancer and revealed that the ISH score of tRF-Pro-AGG-004 and tRF-Leu-CAG-002 in serum can be used as valuable biomarkers for predicting postoperative survival of patients [18]. Together, tsRNAs have important biological functions and potential clinical application in tumors.

In this study, we identified 226 tsRNAs which has significantly differential expression in primary and metastatic pancreatic cancer patients, including 120 upregulated- and 106 downregulated-tsRNAs. Notably, tiRNA-Val-CAC-2 was significantly upregulated in metastatic lesions of pancreatic cancer patients. Overexpression of tiRNA-Val-CAC-2 promoted migration and invasion of pancreatic cancer cells, while knockdown of tiRNA-Val-CAC-2 inhibited these processes. Further mechanistic investigations revealed that tiRNA-Val-CAC-2 could interact with RNA-binding protein FUBP1, leading to the increase of FUBP1 protein stability, which further promotes pancreatic cancer metastasis by *c-MYC* transcription. Taken together, our findings uncovered a novel mechanism in which a tsRNA, tiRNA-Val-CAC-2, regulates pancreatic cancer metastasis, indicating that tiRNA-Val-CAC-2 and FUBP1 could serve as promising therapeutic targets for pancreatic cancer.

## Results

### tsRNAs are differentially expressed between primary and metastatic tissues in pancreatic cancer

**Fig. 1:**
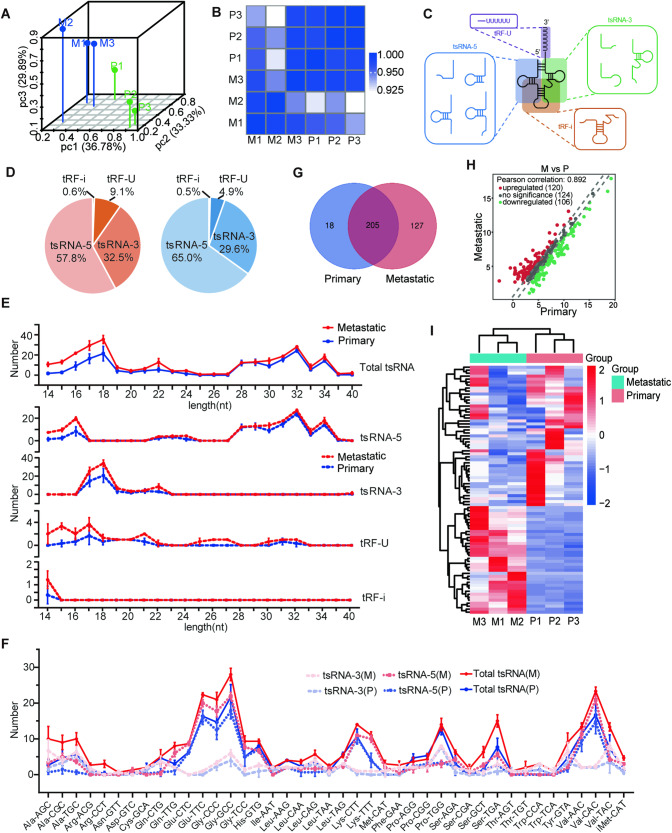
The overview of the tsRNAs expressions in three primary pancreatic cancer tissues and liver metastases tissues of pancreatic cancer. **A** The principal component analysis (PCA) of tsRNAs expressions in primary and metastatic pancreatic cancer samples. M: metastatic pancreatic cancer. P: primary pancreatic cancer. **B** Correlation heatmap analysis of tsRNAs expressions in primary and metastatic pancreatic cancer samples. M: metastatic pancreatic cancer. P: primary pancreatic cancer. **C** Schematic diagram of tsRNA classification. **D** The distribution of tsRNAs subtype numbers in primary (right panel) and metastatic samples (left panel). **E** Length of different tsRNA classifications and number in primary and metastatic samples. **F** The numbers of subtype tsRNAs against tRNA isodecoders in primary and metastatic pancreatic cancer sample. M: metastatic pancreatic cancer. P: primary pancreatic cancer. **F** The distribution of tsRNAs subtype numbers in primary and metastatic samples. **G** Venn diagram based on number of commonly expressed and specifically expressed tsRNAs in primary and metastatic samples. The scatter plot (**H**) between and heatmap (**I**) of tsRNAs in primary and metastatic pancreatic cancer samples. M: metastatic pancreatic cancer; P: primary pancreatic cancer.

To identify and characterize differentially expressed tsRNA, we compared the tsRNA expression profiles between primary tissues and liver metastasis tissues in pancreatic cancer. The clinical information of these samples were showed in Supplementary Table 4. The principal component analysis (PCA) revealed the distinctive expression difference between the primary group and the metastatic group (Fig. 1A). The correlation coefficient analysis indicated a significant correlation between the two groups (Fig. 1B). According to previous research [19], we classified tsRNA into four types, including tsRNA-5, tsRNA-3, tRF-i, tRF-U (Fig. 1C), which account for 57.8%, 32.5%, 9.1%, 0.6% in metastatic group and 65.0%, 29.6%, 4.9%, 0.5% in primary group, respectively (Fig. 1D). Compared with the primary group, more tsRNAs in the range of 14 to 18 nt was detected in the metastatic tumor group. The differential expression of tsRNA between 14 and 16 nt was primarily due to tsRNA-5, while tsRNA-3 accounted for the majority of the differential tsRNA expression between 17 and 18nt (Fig. 1E). We further analyzed the tsRNA produced by different tRNAs. The results revealed that the amount of tsRNA derived from tRNA-Ala-AGC, tRNA-Gln-TTG, tRNA-Lys-TTT, tRNA-Ser-TGA, and tRNA-Val-CAC was higher in the metastatic group than in the primary group. Among the differential tsRNA species, tsRNA-5 was the most prevalent type (Fig. 1F). Subsequent analysis of tsRNA sequencing data revealed the presence of 350 tsRNAs. Of these, 127 and 18 unique tsRNAs were detected in the metastatic and primary groups, respectively (Fig. 1G). The heatmap and scatter plot were utilized to visualize the expression levels and distribution patterns of tsRNAs within the primary and metastatic groups (Fig. 1H, I). As shown in Fig. 1H, a total of 226 differentially expressed tsRNAs in the two groups were identified. Among these, 120 tsRNAs were found to be upregulated, while 106 tsRNAs were downregulated in metastatic groups. The results clearly demonstrated a significant difference in the expression profiles of tsRNAs between primary and metastatic tissues in pancreatic cancer.

### tiRNA-Val-CAC-2 is up‑regulated in metastatic pancreatic cancer

**Fig. 2:**
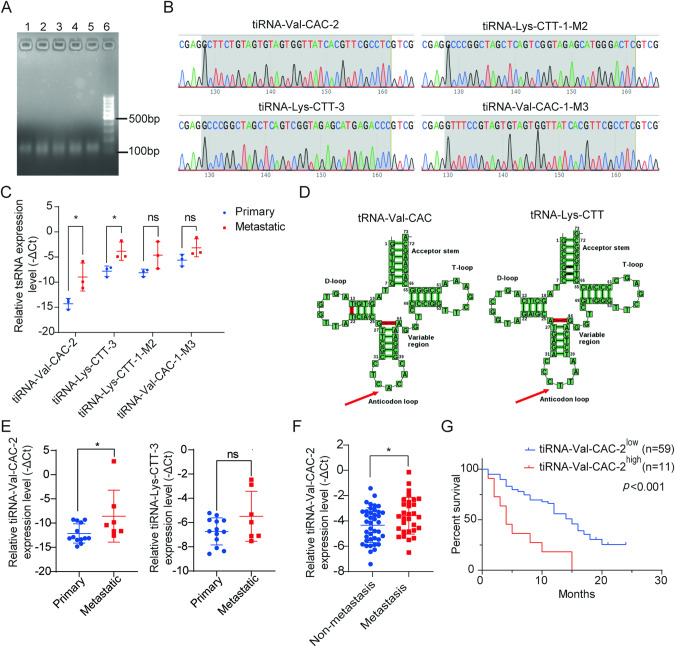
tiRNA-Val-CAC-2 is highly expressed in pancreatic cancer patients with metastatic and is a potential prognostic biomarker for pancreatic cancer. **A** Agarose gel electrophoresis of PCR products of tsRNAs. Lane 1: tiRNA-Val-CAC-2, Lane 2: tiRNA-Lys-CTT-3, Lane 3: tiRNA-Lys-TTT-3-M2, Lane 4: tiRNA-Lys-CTT-1-M2, Lane 5: tiRNA-Val-CAC-1-M3, Lane 6: Marker. **B** Sanger sequencing results of tsRNAs. **C** RT-qPCR was used to analyze the expression of tiRNA-Val-CAC-2, tiRNA-Lys-CTT-3, tiRNA-Lys-CTT-1-M2, and tiRNA-Val-CAC-1-M3 in three primary and metastatic pancreatic cancer samples which were identical to the tsRNA sequencing samples. **D** Cleavage sites of tiRNA-Val-CAC-2 and tiRNA-Lys-CTTT-3 from precursor tRNAs by tRNAdb database. **E** Relative expression of tiRNA-Val-CAC-2 and tiRNA-Lys-CTT-3 in independent primary and metastatic pancreatic cancer tissues. **F** Relative expression of tiRNA-Val-CAC-2 in serum of pancreatic cancer patients with or without metastases. **G** Kaplan–Meier survival analysis of pancreatic cancer patients according to tiRNA-Val-CAC-2 expression in serum of patients. ns, no significance; *p < 0.05.

Accumulative evidence has indicated that tiRNAs, whose cleavage site locates on the anticodon of tRNA, are closely related to tumor development [20]. Aberrantly expressed tiRNAs in human solid tumors may be promising diagnostic biomarkers or therapeutic targets [16, 21, 22]. After taking into account the abundance of tiRNAs (measured in counts per million, CPM), statistical significance (*p*-value < 0.05), and the magnitude of differential expression (|Log2FC| > 1.5), we identified five tiRNAs for further investigation, including tiRNA-Val-CAC-2, tiRNA-Lys-CTT-3, tiRNA-Lys-TTT-3-M2, tiRNA-Lys-CTT-1-M2, and tiRNA-Val-CAC-1-M3 (Supplementary Table 5). Agarose gel electrophoresis was employed to detect the PCR products of the five selected tiRNAs, which displayed a single band of approximately 126 bp in size (Fig. 2A). Subsequently, the products were confirmed by sequencing (Fig. 2B), with the sequences of tiRNA-Val-CAC-2, tiRNA-Lys-CTT-3, tiRNA-Lys-CTT-1-M2, and tiRNA-Val-CAC-1-M3 matching those obtained from tsRNA sequencing. However, the sequence of tiRNA-Lys-TTT-3-M2 did not match that derived from the same samples. Therefore, we proceeded to include the four matched tiRNAs in further RT-qPCR verification. The expression levels of tiRNA-Val-CAC-2 and tiRNA-Lys-CTT-3 were significantly higher in the metastatic samples, as observed from the same RNA samples used for tsRNA sequencing. Conversely, no significant difference was found in the expression levels of tiRNA-Lys-CTT-1-M2 and tiRNA-Val-CAC-1-M3 between the two groups (Fig. 2C). Further analysis using the tRNAdb database (http://trna.bioinf.uni-leipzig.de/DataOutput/Search) confirmed that tiRNA-Val-CAC-2 and tiRNA-Lys-CTT-3 were 5’-tiRNA fragments derived from tRNA-Val-CAC and tRNA-Lys-CTT, respectively (Fig. 2D). Analysis of the mature tRNA sequences revealed that tiRNA-Val-CAC-2 was 34 nt long (5′-GCTTCTGTAGTGTAGTGGTTATCACGTTCGCCTC-3′), with the cleavage site located on the anticodon loop (CAC) (Fig. 2D). Similarly, tiRNA-Lys-CTT-3 was 34 nt in length (5′-GCCCGGCTAGCTCAGTCGGTAGAGCATGAGACCC-3′), with the cleavage site situated on the anticodon loop (CTT) (Fig. 2D). Independent validation of the two tiRNAs demonstrated that the expression of tiRNA-Val-CAC-2 was significantly higher in the metastatic samples (*n* = 7) compared to that in the primary tumors (*n* = 13), while no significant difference was observed in the expression levels of tiRNA-Lys-CTT-3 between these two groups (Fig. 2E).

In addition to tissue samples, further validation of tiRNA-Val-CAC-2 was conducted using independent serum samples, consisting of 40 serum samples from pancreatic cancer patients without distant metastasis and 30 serum samples from pancreatic cancer patients with distant metastasis. The results demonstrated that tiRNA-Val-CAC-2 was expressed at significantly higher levels in the serum of patients with metastases than in those without distant metastasis (Fig. 2F). The association between the serum level of tiRNA-Val-CAC-2 and various clinical characteristics (such as age, gender, tumor stage, tumor grade, tumor size, tumor location, smoking, serum CA199, and serum CEA levels) was evaluated using the chi-square test. The analysis revealed a significant correlation between the level of tiRNA-Val-CAC-2 and age and TNM stage, while no significant correlations were observed with gender, tumor grade, tumor size, tumor location, smoking status, serum CA199, and serum CEA levels (Table 1). Prognostic data from patients with pancreatic cancer were gathered, and the serum level of tiRNA-Val-CAC-2 was classified as either high or low using R language software for prognostic analysis. The results indicated that a high level of tiRNA-Val-CAC-2 in the serum was significantly associated with poor prognosis, as shown in Fig. 2G. Moreover, the Cox proportional hazards model was utilized to conduct univariate analysis, highlighting high levels of tiRNA-Val-CAC-2 (*p* < 0.001) and TNM stage (*p* = 0.006) as significant risk factors influencing clinical survival in pancreatic cancer patients (Table 2). In addition, multivariate analysis was conducted, revealing that both the serum level of tiRNA-Val-CAC-2 (HR = 3.195, *p* = 0.001) and TNM stage (HR = 2.258, *p* = 0.012) were informative prognostic markers for clinical outcomes in patients (Table 3). These findings suggest that tiRNA-Val-CAC-2 could serve as a potential prognostic biomarker in patients with pancreatic cancer.

**Table 1:** Relationship between the level of tiRNA-Val-CAC-2 and clinicopathological parameters of pancreatic cancer patients.

Parameters		*n*	Low expression	High expression	*X*^2^	*p-*value
Number of patients		70	59	11		
Age	≤60	37	28	9	4.393	0.036*
	>60	33	31	2		
Sex	Male	46	39	7	0.025	1.000
	Female	24	20	4		
TNM stage	I–II	20	37	3	4.755	0.045*
	III–IV	50	22	8		
Size	≤3 cm	23	21	2	1.434	0.310
	>3 cm	45	36	9		
	Loss	2				
Location	Head or neck	46	40	6	0.723	0.493
	Body or tail	24	19	5		
Grade	Well and moderate	13	12	1	0.020	1.000
	Poor	22	20	2		
	Loss	35				
Smoke	No	49	39	10	2.516	0.157
	Yes	20	19	1		
	Loss	1				
Preoperative CA19-9 value	<37	13	10	3	0.564	0.428
	≥37	55	47	8		
	Loss	2				
Preoperative CEA value	<5	42	35	7	0.019	1.000
	≥5	26	22	4		
	Loss	2				

*p < 0.05 was considered statistically significant.

**Table 2:** Univariate Cox proportional hazards analysis for survival of pancreatic cancer patients.

Variable	Hazard ratio	95% confidence interval	*p*-value
tiRNA-Val-CAC-2 expression (high/low)	3.513	1.749–7.055	<0.001*
Age (>60/≤60)	0.814	0.472–1.401	0.458
Gender (male/female)	0.843	0.484–1.466	0.545
TNM stage (III–IV/I–II)	2.407	1.280–4.527	0.006*
Grade (poor/moderate and well)	1.628	0.695–3.812	0.262
Size (>3 cm/<3 cm)	1.617	0.887–2.950	0.117
Location (body and tail/head and neck)	1.552	0.892–2.702	0.120
Smoke (yes/no)	0.651	0.353–1.202	0.170
Preoperative CA19-9 value (≥37/<37)	0.587	0.307–1.124	0.108
Preoperative CEA value (≥5/<5)	1.305	0.749–2.273	0.347

*p < 0.05 was considered statistically significant.

**Table 3:** Multivariate Cox proportional hazards analysis for survival of pancreatic cancer patients.

Variables	Hazard ratio	95.0% CI	*p*-value
TNM stage	2.258	1.194–4.272	0.012*
tiRNA-Val-CAC-2 expression	3.195	1.528–6.454	0.001*

*p < 0.05 was considered statistically significant.

### tiRNA-Val-CAC-2 promotes metastasis of pancreatic cancer in vitro and in vivo

**Fig. 3:**
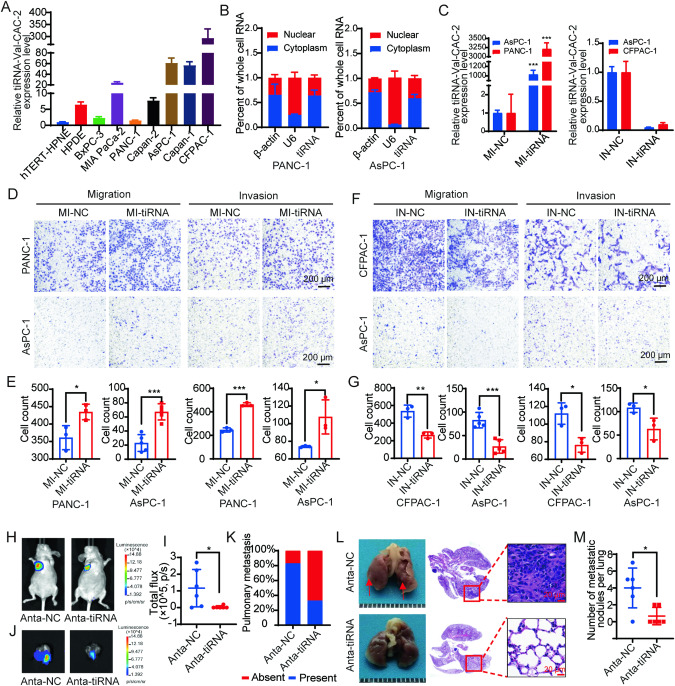
tiRNA-Val-CAC-2 promotes pancreatic cancer metastasis in vitro and in vivo. **A** Relative expression levels of tiRNA-Val-CAC-2 in pancreatic cancer cell lines by RT-qPCR. **B** Localization of tiRNA-Val-CAC-2 in pancreatic cancer cells was determined by a nuclear plasma separation assay. β-actin was used as a cytoplasmic marker and U6 was used as a nuclear marker. Effects of knockdown and overexpression of tiRNA-Val-CAC-2 (**C**) on migration and invasion of PANC-1 (**D**), AsPC-1 (**D**, **F**) and CFPAC-1 (**F**) cells, and responding statistical analysis (**E**, **G**). **H**, **I** Nude mice were transplanted with indicated Luc-labeled CFPAC-1 cells *via* tail vein injection, luciferase activity was visualized 4 weeks post-transplantation (*n* = 6). Lung imaging (**J**) and the proportion of lung with metastases in total six lungs (**K**). **L** Light field map of lung tumor nodules (left panel) and HE staining of lung tissue (right panel). **M** Statistics on the number of tumor nodules per lung. MI-NC: mimic NC; MI-tiRNA: mimic tiRNA-Val-CAC-2; IN-NC: inhibitor NC; IN-tiRNA: inhibitor tiRNA-Val-CAC-2; Anta-NC antagomir NC, Anta-tiRNA antagomir tiRNA-Val-CAC-2. *p < 0.05, **p < 0.01, ***p < 0.001.

To investigate the role of tiRNA-Val-CAC-2 in pancreatic cancer, we first examined its expression and localization in pancreatic cancer cell lines. RT-qPCR results showed that the expression of tiRNA-Val-CAC-2 in most pancreatic cancer cells was higher than that in normal pancreatic ductal epithelial cells (hTERT-HPNE and HPDE) (Fig. 3A). To investigate the localization of tiRNA-Val-CAC-2 in cells, we employed nucleoplasmic separation assay and found that tiRNA-Val-CAC-2 was present in both the cytoplasm and nucleus of PANC-1 cells and AsPC-1 cells (Fig. 3B).

Subsequently, we synthesized mimic and inhibitor of tiRNA-Val-CAC-2 in order to investigate its impact on pancreatic cancer cell metastasis. Results of migration and invasion assays showed that overexpression of tiRNA-Val-CAC-2 significantly augmented the migration and invasion capabilities of pancreatic cancer cells. Conversely, knockdown of tiRNA-Val-CAC-2 led to a reduction in the migration and invasion abilities of pancreatic cancer cells (Fig. 3C–G).

Furthermore, we explored the effect of tiRNA-Val-CAC-2 on pancreatic cancer cell metastasis in vivo utilizing a mouse lung metastasis model. Specifically, antagomir NC and antagomir tiRNA-Val-CAC-2 were transfected into CFPAC-1 cells, respectively, and then intravenously injected into nude mice. The progression of lung metastasis was subsequently monitored by utilizing an in vivo imaging system. In vivo bioluminescence imaging demonstrated that both the fluorescence intensity in the lungs and the number of metastatic lungs were significantly lower in the antagomir tiRNA-Val-CAC-2 group than in the control group (Fig. 3H–K). Moreover, tiRNA-Val-CAC-2 knockdown significantly decreased the number of metastatic nodules in the lungs (Fig. 3L, M). These results suggest that tiRNA-Val-CAC-2 plays a key role in the promotion of pancreatic cancer metastasis in vivo.

### tiRNA-Val-CAC-2 interacts with FUBP1

**Fig. 4:**
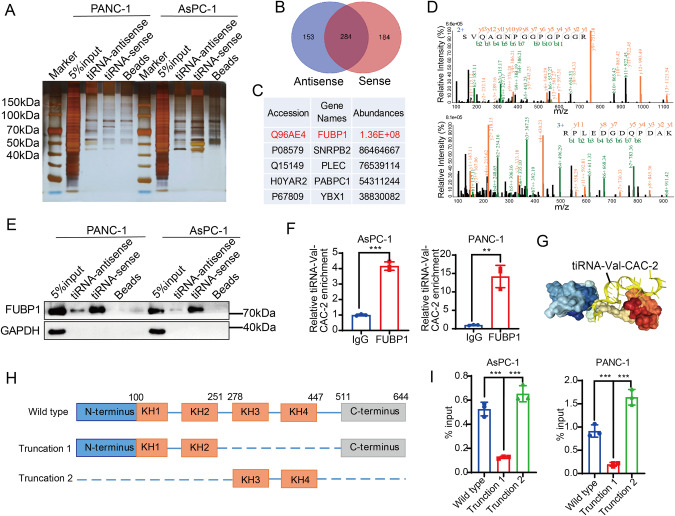
tiRNA-Val-CAC-2 interacts with RNA-binding protein FUBP1. **A** Silver staining showing affinity capture of proteins from PANC-1 and AsPC-1 cells by RNA pull down. **B** Venn diagram of mass spectrometry results of PANC-1 cells from (**A**). **C** Top 5 abundant proteins in the sense strand group from mass spectrum. **D** Representative peptide of FUBP1 protein from mass spectrum. **E** FUBP1 protein enrichment in RNA pull down samples by western blot. **F** RIP and RT-qPCR assay were used to test the enrichment of FUBP1 on tiRNA-Val-CAC-2 in AsPC-1 and PANC-1 cells. IgG was used as a negative control. **G** The binding pattern of tiRNA-Val-CAC-2 and FUBP1 protein predicted by HDOCK database. tiRNA-Val-CAC-2 is shown in yellow, which is predicted to bind to the KH3-KH4 domain of FUBP1. **H** Diagram of the full-length and truncated structures of FUBP1. **I** Enrichment of full-length and truncated FUBP1 in tiRNA-Val-CAC-2 was determined by q-PCR after RIP assay. tiRNA-sense: tiRNA-Val-CAC-2; tiRNA-antisense: reverse complementary sequence of tiRNA-Val-CAC-2. *p < 0.05, **p < 0.01, ***p < 0.001.

To further explore the underlying mechanism by which tsRNA regulates the metastasis, we utilized RNA pull-down and mass spectrometry assays to identify tiRNA-Val-CAC-2 associated proteins. We performed RNA pull-down assay using biotin-labeled tiRNA-Val-CAC-2 and the resulted proteins were detected by silver staining. As shown in Fig. 4A, there was a distinct band in the 70-100 kDa position in the sense strand of both cell lines. Meanwhile, we identified 184 unique proteins in the sense strand group of RNA pull-down samples by mass spectrometry analysis (Fig. 4B). Among these proteins, FUBP1 was identified as the most significant protein, based on the ranking of protein abundance (Fig. 4C). Additionally, two unique major peptides of FUBP1 were detected, as depicted in Fig. 4D. To further validate the mass spectrometry results, we conducted western blot and observed that tiRNA-Val-CAC-2 sense strand was indeed able to pull down more FUBP1 protein compared to the antisense strand (Fig. 4E). Further, we also confirmed the binding between FUBP1 and tiRNA-Val-CAC-2 by RNA immunoprecipitation assay. As shown in Fig. 4F, the results demonstrated that FUBP1 could substantially enrich tiRNA-Val-CAC-2, indicating the interaction between FUBP1 and tiRNA-Val-CAC-2. To gain insight of the binding pattern between tiRNA-Val-CAC-2 to FUBP1, we employed the online database HDOCK (Fig. 4G), and the detailed binding sites of tiRNA-Val-CAC-2 to FUBP1 amino acids were also predicted and shown in supplementary Table 6. These results suggested that tiRNA-Val-CAC-2 mainly binds to the KH3-KH4 domain of FUBP1. In order to validate this prediction, we constructed a wild-type FUBP1 overexpression plasmid, as well as truncated plasmids of FUBP1 lacking the KH3-KH4 domain or only including KH3-KH4 domain as shown in Fig. 4H. RIP experiments were then performed in wild-type and/or mutant FUBP1 transfected AsPC-1 and PANC-1 cells using FLAG antibodies. Results clearly showed that deletion of the KH3-KH4 domain significantly reduced the binding of tiRNA-Val-CAC-2 to FUBP1 (Fig. 4I), indicating that tiRNA-Val-CAC-2 mainly interacts with KH3-KH4 domain of FUBP1 protein.

### tiRNA-Val-CAC-2 increases FUBP1 protein stability

**Fig. 5:**
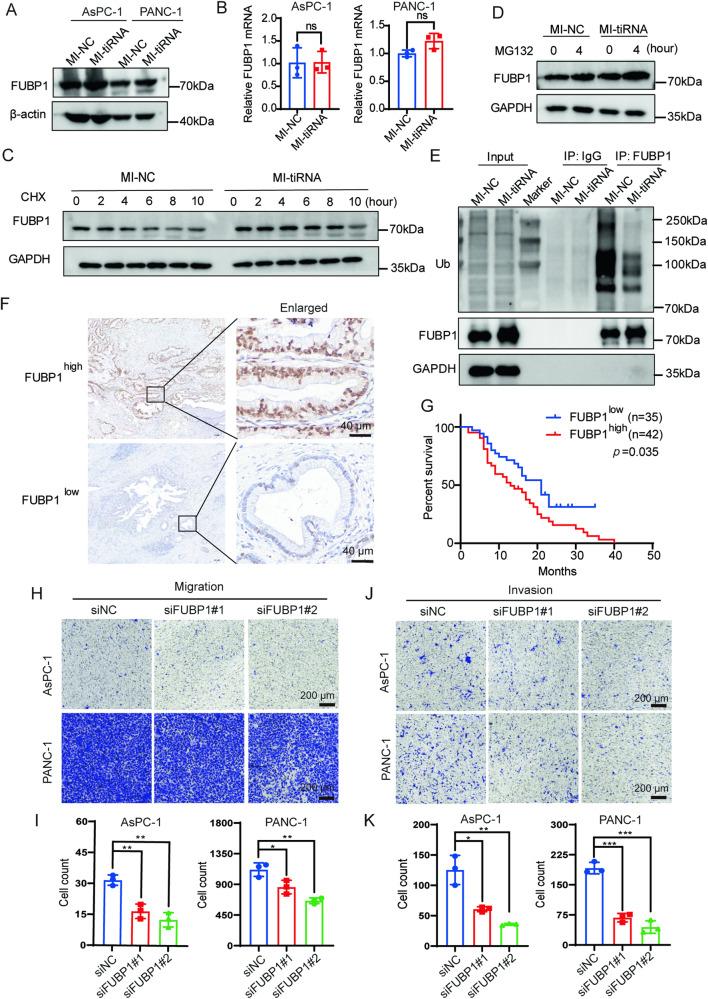
tiRNA-Val-CAC-2 promotes the stability of FUBP1 protein which can be used as a prognostic biomarker. **A** The protein level of FUBP1 was detected by western blot after overexpression of tiRNA-Val-CAC-2 in AsPC-1 and PANC-1 cells. **B** The mRNA level of FUBP1 was detected by RT-qPCR after overexpression of tiRNA-Val-CAC-2 in AsPC-1 and PANC-1 cells. **C** After the AsPC-1 cells were treated with 100 μg/mL Cycloheximide (CHX), the half-life of FUBP1 protein was detected by western blot. **D** After AsPC-1 cells were treated with 10 μM MG132, the effect of tiRNA-Val-CAC-2 on the degradation of FUBP1 protein was detected by western blot. **E** The ubiquitination level of FUBP1 protein was detected by western blot after overexpression of tiRNA-Val-CAC-2 in AsPC-1 cell. **F** Representative immunohistochemical images of FUBP1 high and low expression in pancreatic cancer tissues (*n* = 77). **G** Kaplan–Meier survival analysis of pancreatic cancer patients according to FUBP1 expression. (H-K) Knockdown of FUBP1 inhibited the migration (**H**, **I**) and invasion (**J**, **K**) of pancreatic cancer cells. mimic NC; MI-tiRNA: mimic tiRNA-Val-CAC-2. *p < 0.05, **p < 0.01, ***p < 0.001.

To further explore the impact of tiRNA-Val-CAC-2 on FUBP1, we overexpressed tiRNA-Val-CAC-2 in PANC-1 and AsPC-1 cells, and detected FUBP1 expression (Fig. 5A). Interestingly, despite observing an increase of FUBP1 protein upon overexpression of tiRNA-Val-CAC-2, there was no obvious change in its mRNA level (Fig. 5B). And the loss-of-function experiments also indicated that tiRNA-Val-CAC-2 knockdown decreased the FUBP1 protein other than its mRNA (Supplementary Fig. 1A- B). These results prompted us to speculate that tiRNA-Val-CAC-2 may exert its effect on FUBP1 by influencing its protein stability, rather than affecting transcription. To test this possibility, we treated tiRNA-Val-CAC-2 overexpressing or knockdown cell (AsPC-1) with protein synthesis inhibitor cycloheximide (CHX) and 26S proteasome inhibitor MG132 and collected cell lysates for western blot. As illustrated in Fig. 5C, the treatment with CHX led to a remarkable extension in the half-life of FUBP1 protein in the tiRNA-Val-CAC-2 overexpression group compared to the control group, while tiRNA-Val-CAC-2 knockdown reduced the FUBP1 protein half-life (Supplementary Fig. 1C). In addition, the inhibition of 26S proteasome with MG132 resulted in a noticeable increase of FUBP1 protein in the tiRNA-Val-CAC-2 overexpression group, as compared to the control group (Fig. 5D), while tiRNA-Val-CAC-2 knockdown decreased FUBP1 protein expression (Supplementary Fig. 1D). We further examined the effect of tiRNA-Val-CAC-2 on FUBP1 protein ubiquitination. Co-IP assay in AsPC-1 cell showed that overexpression of tiRNA-Val-CAC-2 reduced the ubiquitination level of FUBP1 (Fig. 5E). These results imply that tiRNA-Val-CAC-2 could regulate the stability of FUBP1 protein by decreasing the degradation *via* ubiquitination.

### FUBP1 is a potential prognostic biomarker of pancreatic cancer

To explore the role of FUBP1 in pancreatic cancer, immunohistochemical staining was performed on 77 cases of pancreatic cancer. The staining results were divided into high and low expression groups (Fig. 5F). Based on the staining results, we categorized the samples into groups with either high or low expression levels (Fig. 5F). Clinical features and survival data of these patients were also collected and analyzed for potential correlations. The results presented in Table 4 indicated a significant relationship between FUBP1 expression and the tumor grade of pancreatic cancer patients. Specifically, samples from patients with poorly differentiated tumors demonstrated higher FUBP1 expression. However, FUBP1 expression did not exhibit any correlation with patient gender, age, TNM stage, presence of neural invasion, or serum CA199 and CEA expression levels. It is noteworthy that patients with high FUBP1 expression displayed a poorer prognosis compared to those with low FUBP1 expression (Fig. 5G). In addition, univariate analysis with the Cox proportional hazards model identified both high FUBP1 level (HR = 1.771, *p* = 0.044) and tumor grade (HR = 2.090, *p* = 0.013) as statistically significant risk factors influencing the clinical survival of pancreatic cancer patients (Table 5). These results indicate that FUBP1 can serve as a potential prognostic biomarker for individuals diagnosed with pancreatic cancer. To investigate the impact of FUBP1 on pancreatic cancer cell metastasis, we utilized transwell migration and invasion assays in FUBP1 knockdown pancreatic cancer cells and observed that the obvious reduction in migration and invasion (Fig. 5H–K), indicating that FUBP1 indeed regulates the metastasis of pancreatic cancer.

**Table 4:** Relationship between FUBP1 expression and clinicopathological parameters of pancreatic cancer patients.

Parameters		*n*	Low expression	High expression	*X*^2^	*p-*value
Number of patients		77	35	42		
Age	≤60	36	17	19	0.085	0.770
	>60	41	18	23		
Sex	Male	43	20	23	0.044	0.834
	Female	34	15	19		
TNM stage	I–II	74	34	40	0.185	1.000
	III–IV	3	1	2		
Lymph node metastasis	No	53	25	28	0.202	0.653
	Yes	24	10	14		
Grade	Well and moderate	26	17	9	6.289	0.012*
	Poor	51	18	33		
Nerve invasion	Yes	60	28	32	0.161	0.688
	No	17	7	10		
Preoperative CA19-9 value	<37	21	8	13	0.631	0.427
	≥37	56	27	29		
Preoperative CEA value	<5	55	23	32	1.027	0.311
	≥5	22	12	10		

*p < 0.05 was considered statistically significant.

**Table 5:** Univariate Cox proportional hazards analysis for survival of pancreatic cancer patients.

Variable	Hazard ratio	95% confidence interval	*p*-value
FUBP1 expression (high/low)	1.771	1.017–3.068	0.044*
Age (≤60/>60)	1.359	0.792–2.331	0.265
Gender (female/male)	0.853	0.501–1.452	0.558
TNM stage (III–IV/I–II)	0.210	0.028–1565	0.128
Grade (poor/moderate and well)	2.090	1.169–3.736	0.013*
Nerve invasion (no /yes)	1.495	0.786–2.845	0.221
Preoperative CA19-9 value (≥37/<37)	1.552	0.845–2.852	0.157
Preoperative CEA value (≥5/<5)	0.670	0.365–1.232	0.198

*p < 0.05 was considered statistically significant.

### tiRNA-Val-CAC-2 promotes metastasis of pancreatic cancer by up-regulating *c-MYC* transcription through FUBP1

**Fig. 6:**
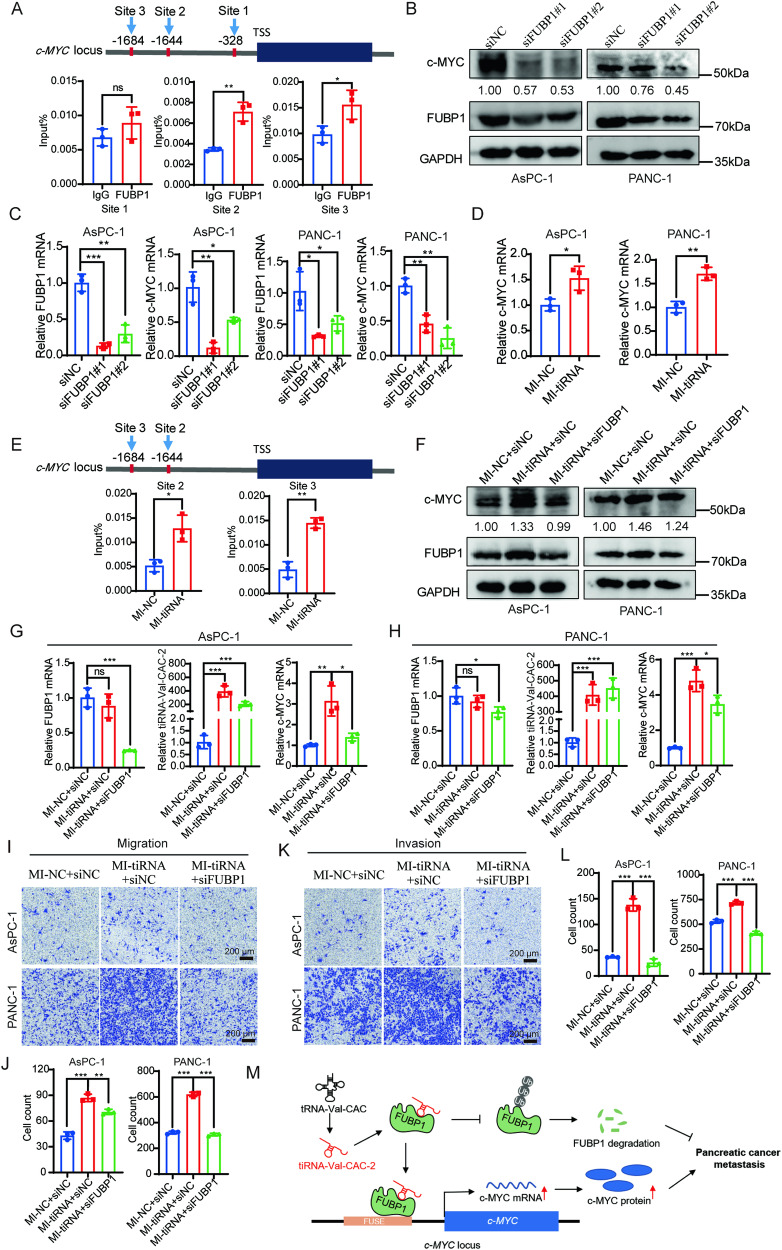
tiRNA-Val-CAC-2 facilitates pancreatic cancer metastasis by up-regulating *c-MYC* transcription through interaction with FUBP1. **A** Chromatin immunoprecipitation (ChIP) analysis of FUBP1 enrichment on upstream of *c-MYC* transcription start site in AsPC-1 cells. FUBP1 knockdown inhibited the expression of c-MYC protein (**B**) and mRNA (**C**) in AsPC-1 and PANC-1 cells by western blot and RT-qPCR assay. **D** tiRNA-Val-CAC-2 overexpression increased the expression of c-MYC mRNA in AsPC-1 and PANC-1 cells by RT-qPCR assay. **E** ChIP analysis of FUBP1 enrichment on FUSE element of *c-MYC* in AsPC-1 cells after tiRNA-Val-CAC-2 overexpression. The protein (**F**) and mRNA (**G**, **H**) expression of c-MYC was measured in control, tiRNA-Val-CAC-2 overexpression, and rescue groups by western blot and RT-qPCR. The migration (**I**, **J**) and invasion (**K**, **L**) abilities were measured in control, tiRNA-Val-CAC-2 overexpression, and rescue groups by transwell assay. **M** Schematic diagram of tiRNA-Val-CAC-2 promoting metastasis in pancreatic cancer. By interacting with FUBP1, tiRNA-Val-CAC-2 maintained the stability of FUBP1, increased FUBP1 enrichment in the FUSE region of c-MYC gene, promoted the transcription of proto-oncogene c-MYC, and eventually led to pancreatic cancer metastasis. FUSE: far-upstream element sequence. MI-NC: mimic NC; MI-tiRNA: mimic tiRNA-Val-CAC-2. ns no significance; *p < 0.05, **p < 0.01, ***p < 0.001.

Previous studies have reported that FUBP1 protein can regulate *c-MYC* transcription by binding to the *c-MYC* far-upstream element (FUSE) sequence, thereby affecting tumor metastasis in lung cancer and breast cancer [23, 24]. To evaluate the potential impact of FUBP1 on *c-MYC* transcription in pancreatic cancer cells, we performed ChIP assays using specific antibody against FUBP1. Results showed that FUBP1 was not substantially enriched in the *c-MYC* promoter region located near the transcription start site (Site 1, Fig. 6A). However, FUBP1 was significantly enriched in the FUSE region (Site 2 and Site 3, Fig. 6A). We further observed a reduction in c-MYC expression levels in FUBP1-knockdown pancreatic cancer cells (Fig. 6B, C). Taken together, these results suggest that FUBP1 may play a critical role in the regulation on c-MYC transcription in pancreatic cancer probably through interaction with FUSE motif.

Based on the aforementioned findings, we speculated that tiRNA-Val-CAC-2 may be involved in FUBP1-mediated *c-MYC* transcription. To test this hypothesis, we firstly measured c-MYC mRNA levels by RT-qPCR and found that overexpression of tiRNA-Val-CAC-2 is able to increase *c-MYC* transcription (Fig. 6D). Next, we performed ChIP assay in cells overexpressing tiRNA-Val-CAC-2 and found that tiRNA-Val-CAC-2 promotes the transcription of *c-MYC* by binding to the *c-MYC* FUSE region through FUBP1 (Site 2 and Site 3, Fig. 6E). Since tiRNA-Val-CAC-2 is capable of promoting FUBP1-mediated *c-MYC* transcription in pancreatic cancer cells, we further hypothesized that tiRNA-Val-CAC-2 may promote metastasis in pancreatic cancer through FUBP1 to active *c-MYC* transcription. To verify this possibility, we conducted rescue experiments in AsPC-1 and PANC-1 cells and observed an increase in both the protein levels of c-MYC and FUBP1 following the overexpression of tiRNA-Val-CAC-2 (Fig. 6F). Of note, we found that both protein level and mRNA level of c-MYC in the rescue group were successfully restored by FUBP1 knockdown when compared to overexpression tiRNA-Val-CAC-2 group (Fig. 6F–H). Additionally, we also found that overexpression of FUBP1 successfully reversed c-MYC protein and mRNA levels in the rescue group as compared with the tiRNA-Val-CAC-2 knockdown group (Supplementary Fig. 2A, B). Furthermore, the results from transwell migration and invasion assays revealed that FUBP1 knockdown attenuate the ability of tiRNA-Val-CAC-2 in promoting cancer cell migration and invasion (Fig. 6I–L). As we expected, FUBP1 can improve the reduced ability of migration and invasion caused by tiRNA-Val-CAC-2 knockdown (Supplementary Fig. 2C, D). These findings provide further evidence in support of notion that tiRNA-Val-CAC-2 promotes the metastasis through the FUBP1-mediated activation of *c-MYC* transcription in pancreatic cancer (Fig. 6M).

**Fig. 7:**
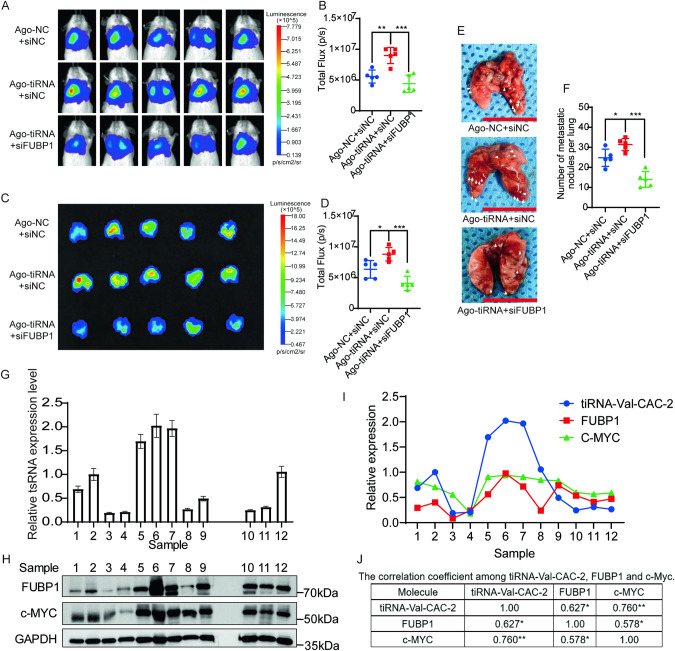
tiRNA-Val-CAC-2 facilitates pancreatic cancer metastasis by FUBP1 in vivo, and the correlation among tiRNA-Val-CAC-2, FUBP1 and c-MYC. **A**, **B** Mice were transplanted with indicated Luc-labeled PANC-1 cells *via* tail vein injection, luciferase activity was visualized 20-days post-transplantation (*n* = 5). **C**, **D** Lung imaging and total fluorescence statistics. **E**, **F** Light field of lung tumor nodules and statistics on the number of tumor nodules per lung. Scar bar=1 cm. **G** The expression of tiRNA-Val-CAC-2 in tumor tissues from 12 patients with pancreatic cancer via RT-qPCR. **H** Western blot was used to detect the protein expression of FUBP1 and c-MYC in tumor tissues from 12 patients with pancreatic cancer. **I** The expression of tiRNA-Val-CAC-2, FUBP1 and c-MYC in tumor tissues from 12 patients with pancreatic cancer by quantitative analysis. **J** The correlation coefficient among tiRNA-Val-CAC-2, FUBP1 and c-Myc. Ago-NC: agomir NC; Ago-tiRNA: agomir tiRNA-Val-CAC-2; *p < 0.05, **p < 0.01, ***p < 0.001.

To investigate the potential influence of tiRNA-Val-CAC-2 on pancreatic cancer metastasis through FUBP1 in vivo, we conducted rescue animal experiments. The result revealed that tiRNA-Val-CAC-2 overexpression significantly promoted lung metastasis of PANC-1 cells in vivo. However, when FUBP1 was knocked down, the metastatic effect induced by tiRNA-Val-CAC-2 was counterbalanced (Fig. 7A–F). Furthermore, we investigated the association between tiRNA, FUBP1, and c-MYC using tumor samples obtained from 12 pancreatic cancer patients. Remarkably, we observed a consistent expression pattern of these three factors in most of the tumor patients (Fig. 7G–I). Moreover, a statistically significant correlation was found among them (Fig. 7J). Taken together, our findings unearthed differences in tsRNA expression between primary and metastatic pancreatic cancer, as well as highlighted the high abundance of a novel tiRNA, referred to as tiRNA-Val-CAC-2. Knockdown of tiRNA-Val-CAC-2 restricted the metastatic potential of pancreatic cancer cells. Mechanistically, tiRNA-Val-CAC-2 interacts with the KH3-KH4 domain of FUBP1 to inhibit FUBP1 degradation. Thus, in turn, it leads to the accumulation of FUBP1 protein at the far-upstream element (FUSE) of the *c-MYC* proto-oncogene, which facilitates *c-MYC* transcription and ultimately results in the metastasis (Fig. 6M). Importantly, we also discovered that tiRNA-Val-CAC-2 is highly expressed in the serum of pancreatic cancer patients with metastasis, high levels of tiRNA-Val-CAC-2 are closely related with poor prognosis. These findings shed light on the potential of tiRNA-Val-CAC-2 serving as a prognostic biomarker and therapeutic target for pancreatic cancer.

## Discussion

tsRNAs were originally believed to be random degradation products of tRNAs. However, it is now widely recognized that the expression of certain tsRNAs is formed by specific selective cleavage of tRNAs under certain stress, which relies on specific enzymes such as angiogenin (ANG), RNase T2, and RNase L [25, 26]. Therefore, it is necessary to distinguish between small RNAs generated solely from the degradation of longer transcripts and functional small RNAs. In this study, we observed a significant presence of tsRNAs in the size range of 16–18 nt and 31-33 nt through tsRNA sequencing. This observation aligns with previous research conducted by Cole et al. [27], which reported similar length patterns for tsRNA-5 (32–33 nt) and tsRNA-3 (16–18 nt). Additionally, we noticed a distinct peak of tsRNA-5 in the 14–16 nt range. Due to the construction and sequencing of small RNA libraries often generating fragments from various non-coding RNAs such as ribosomal RNA, transfer RNA, small nucleolar RNA, and small nuclear RNAs [27], it cannot be ruled out that the presence of tsRNA-5 in the 14-16 nt might be attributed to tRNA degradation processes.

Cancer remains the leading cause of mortality globally, with poor prognoses for many patients due to the absence of reliable early diagnostic markers and effective anti-cancer treatments. However, the emergence of tsRNAs as a novel class of small non-coding RNAs has opened up new perspectives for the development of diagnostic biomarkers and therapeutic targets for cancer. For example, Wu et al. found that the combination of plasma 5’-TRF-Gly-GCC with carcinoembryonic antigen and carbohydrate antigen 199 in colon cancer patients increased the AUC value to 0.926 [28]; Importantly, Panoutsopoulou et al. found a significant correlation between i-tRF-GlyGCC and early progression as well as poor overall survival [29]; In addition, Li et al. discovered that the expression level of serum tRF-29-R9J8909NF5JP is significantly elevated in gastric cancer tissues, and its high expression is associated with lower survival rates [30]. In this study, we found that the expression of tiRNA-Val-CAC-2 is significantly upregulated in metastatic lesions of pancreatic cancer compared to primary lesions, and patients with high expression of serum tiRNA-Val-CAC-2 demonstrated a poorer prognosis. Furthermore, we found that tiRNA-Val-CAC-2 plays a critical role in promoting metastasis in pancreatic cancer. Of note, Mo et al. reported that 5′-tiRNA-Val acts as a potential tumor-suppressor *via* FZD3-mediated Wnt/ beta-catenin signaling pathway in the progression of breast cancer [15]. Regarding the different roles of the two tsRNAs derived from the same mature tRNA in tumor, there are some potential explanations. Firstly, the sequence and size of the two tsRNAs are not exactly the same. Secondly, the mechanism of action of 5′-tiRNA-Val discovered by Mo et al. is completely different from that of tiRNA-Val-CAC-2 found by our study. Alternatively, it may be caused by different tumor types. Many studies have found that the same gene may play different functions in different tumors, and some even are completely opposite. For example, ID4 gene is typically highly expressed in normal prostate tissue but exhibits decreased expression in prostate cancer tissue and gradually decreases with cancer progression, highlighting the conventional inactivation pattern of a tumor-suppressor gene. However, the ID4 gene may act as a proto-oncogene, either being highly expressed in tumor tissue or exhibiting an increased copy number in bladder cancer [31]. Of course, other more plausible explanations are need to be further explored.

In addition, recent studies have shown that tsRNAs can be present in extracellular vesicles, such as exosomes, in the blood. For example, Zhu et al. observed the widespread presence of tsRNAs in exosomes and identified four tsRNAs that are significantly elevated in the plasma of liver cancer patients [32]. However, our study examined the total tsRNA in the serum, and whether tsRNAs exist in the form of extracellular vesicles, specifically exosomes, in the serum requires further exploration.

Different tsRNAs have distinct functions and mechanisms of action. Chen et al. summarized three primary mechanisms of tsRNAs from an evolutionary perspective, including tRNA mimicry/displacement, formation of tsRNA-ribonucleoproteins complexes, and association with Argonaute proteins [33]. Yu et al. concluded that tsRNAs play important roles in RNA silencing, translation regulation, and epigenetic modulation [34]. For example, tRF-Val directly binds to EEF1A1, facilitating the transport of EEF1A1 to the nucleus and enhances its interaction with MDM2, ultimately resulting in the inhibition of the downstream molecular pathways of P53 and promoting gastric cancer progression [35]. AS-TDR-007333 activates *MED29* to further promote malignant transformation of non-small cell lung cancer cells through two different mechanisms. AS-TDR-007333 interacts with HSPB1 to enhance the H3K4me1 and H3K27ac in *MED29* promoter to activate *MED29* expression. Additionally, AS-TDR-007333 triggers an increase in the expression of transcription factor ELK4, which binds to the *MED29* promoter and further promotes its transcriptional activity [36]. Using RNA pull down and mass spectrometry analysis, we identified that tiRNA-Val-CAC-2 can interact with RNA-binding protein FUBP1. FUBP1 is a type of fusion binding protein that has the ability to recognize single-stranded DNA, and it belongs to the FUBP protein family [37, 38]. Previous studies have reported that FUBP1 is capable of promoting epithelial-mesenchymal transition of pancreatic cancer cells through the TGF-β/Smad pathway [39]. Nevertheless, the precise mechanisms of FUBP1 in pancreatic cancer remain elusive. Additionally, evidence suggests that FUBP1 can bind to non-coding RNAs and play a crucial role in tumor development. For example, FUBP1 can be recruited to *c-MYC* FUSE through lncRNA binding, resulting in the activation of *c-MYC* transcription and facilitating the proliferation, survival, invasion, and metastasis of lung cancer cells [23]. Additionally, FUBP1 can be competitively bound by circACTN4, resulting in reduced FIR-binding and subsequently triggering the activation of *c-MYC* transcription, which leads to the progression of breast cancer [24]. In this study, we revealed that tiRNA-Val-CAC-2 interacts specifically with the KH3-KH4 domains of FUBP1, leading to increased stabilization of the FUBP1 protein and its subsequent enrichment in *c-MYC* transcriptional regulation. Thus, in turn, it contributed to the transcriptional activation of *c-MYC*. It’s worth mentioning that our findings indicated that tiRNA-Val-CAC-2 has the potential capacity to inhibit the ubiquitination of FUBP1 protein. As such, it is hypothesized that tiRNA-Val-CAC-2 disrupts the interaction between FUBP1 and related ubiquitin ligases in pancreatic cancer. Despite this, the specific E3 ubiquitin ligases that target FUBP1 remain elusive and require further investigation for future studies.

## Materials and methods

Experimental procedures are provided in the Supplementary Materials.

### Supplementary information

Supplementary materials

## Data Availability

All data and materials were available from the corresponding authors on reasonable request. The tsRNA sequencing data have been uploaded in GEO DataSets (GSE251943).
