# Supplementary material for: tiRNA-Val-CAC-2 interacts with FUBP1 to promote pancreatic cancer metastasis by activating c‑MYC transcription
Source: Oncogene. 2024 Mar 5;43(17):1274–87. doi: 10.1038/s41388-024-02991-9 (PMC11035144; doi:10.1038/s41388-024-02991-9)
Supplement: Supplementary file 1 — Supplementary materials [file 41388_2024_2991_MOESM1_ESM.docx]

**Supplementary Materials**

**Materials and Methods**

**Clinical samples**

All primary and metastatic tissues of pancreatic cancer were obtained *via* surgical resection from patients who underwent gastrectomy at West China Hospital, Sichuan University between 2020 and 2021 and were subsequently preserved in liquid nitrogen. Among these tissues, three cases of primary pancreatic cancer and three cases of metastatic tumors were subjected to tsRNA sequencing, while the remaining samples were used for tsRNA validation. Blood samples were collected from pancreatic cancer patients with or without metastasis, with informed consent obtained from each patient. All pancreatic cancer diagnoses were confirmed *via* histopathological analysis, and tumor staging was based on the tumor-node-metastasis (TNM) system (Gabriel, Stephen, & Armando, 2018). Whole-blood samples (10 mL) were collected before surgery into purple-top tubes, with plasma prepared within 1 hour of collection by centrifugation at 1300×g for 10 minutes at 4°C and stored at -80°C for further experiments. The entire sample processing procedure was performed on ice, and informed consent was obtained from each patient in accordance with committee policies. The study was conducted in accordance with the Declaration of Helsinki and approved by the Ethics Committee of West China Hospital, Sichuan University.

**The tsRNA sequencing and analysis procedure**

The rtStar™ tRF&tiRNA Pretreatment Kit (Arraystar) was utilized to eliminate the interfering modifications that hamper small RNA-seq library construction or qPCR. This kit encompasses the necessary reagents to perform specific treatments on total RNA samples before library preparation, including: 3′-aminoacyl (charged) deacylation to convert it to 3′-OH for 3′ adaptor ligation, 3′-cP (2′,3′-cyclic phosphate) removal to convert it to 3′-OH for 3′ adaptor ligation, 5′-OH (hydroxyl group) phosphorylation to convert it to 5′-P for 5′-adaptor ligation, and m1A and m3C demethylation to facilitate efficient reverse transcription. Furthermore, for qPCR detection, the rtStar™ First-Strand cDNA Synthesis Kit (5′ and 3′ Adaptors) (Arraystar) was adopted to create cDNA libraries from small RNAs following the manufacturer’s guidelines. This kit provides the requisite reagents to consecutively ligate 3′-Adaptor with its 5′-end to the 3′-end of the RNAs and 5′-Adaptor with its 3′-end to the 5′-end of the RNAs. The non-ligation ends of 3′ and 5′ Adaptors are modified to prevent ligation. The 3′ Adaptor carries a universal priming site for Reverse Transcription (RT) Primer.

Raw sequencing data generated form Illumina NextSeq 500 that pass the Illumina chastity filter are used to the following analysis. Trimmed reads (trimmed 5’, 3’-adaptor bases) are aligned allowing for 1 mismatch only to the mature tRNA sequences, then reads that do not map are aligned allowing for 1 mismatch only to precursor tRNA sequences with bowtie software. The expression profiling of tsRNAs is calculated based on counts of reads mapped. The differentially expressed tsRNAs are screened based on the count value with R package edgeR.

**Quantitative real-time polymerase chain reaction (qRT-PCR) and PCR**

The total RNAs obtained from human pancreatic cancer tissues, cell lines, and plasma samples were extracted utilizing the Trizol reagent (Thermo). For reverse transcription of tsRNAs, the miRNA 1st Strand cDNA Synthesis Kit (Vazyme) was employed, following the manufacturer's instructions. The cDNA amplification was conducted utilizing the SYBR miRNA Premix (Vazyme) in accordance with the manufacturer's protocol for qRT-PCR. HiScript III RT SuperMix for qPCR (Vazyme) was utilized for the reverse transcription of mRNA. ChamQ SYBR qPCR Master Mix (Vazyme) was applied for performing real-time quantitative PCR. The internal control for tsRNA was U6, while GAPDH was used as the internal control for mRNA. The −ΔCt or 2-ΔΔCt method was utilized for the relative quantification of expression to calculate the levels of tsRNA. Moreover, 2−△△Ct method was used for the normalization of related mRNA target gene expression against GAPDH. All primers used in the study were listed in Supplementary Table 1. For PCR assay, we used the products of reverse transcription as the template, and the PCR primers used were listed in Supplementary Table 1.

**Cell culture**

The AsPc-1, BxPC-3, Capan-1, Capan-2, CFPAC-1, MIA PaCa-2, PANC-1, HPDE and hTERT-HPNE cell lines used in this study were obtained from the Cell Bank of the Shanghai Institute of Cells, Chinese Academy of Science or BeNa Culture Collection. The cells were cultured in Dulbecco Modified Eagle Medium (HyClone), 1640 medium (HyClone), or IMDM medium (Gibco) supplemented with 10% fetal bovine serum (Excell), as well as 100 U/mL penicillin G and 100 mg/mL streptomycin (Beyotime), and maintained in a humidified 5% CO2 incubator at 37 °C.

**Cell transfection**

In this study, tsRNA mimic, inhibitor and corresponding negative control (NC) were synthesized by Sangon Biotech (Shanghai, China). The tsRNA oligonucleotides were transfected using Lipofectamine 3000 (Invitrogen) in Reduced Serum Media (Invitrogen). The transfection medium was refreshed 6 hours post transfected, and cells were incubated for 48 hours for subsequent *in vitro* studies. To investigate the potential regulatory effect of FUBP1 on tsRNA, small interfering RNAs against FUBP1 (siFUBP1) and the negative control (siNC) were designed and synthesized by Youkangjianxing Biotechnology company (Chengdu, China). Lipofectamine 3000 reagent (Invitrogen) was used as the transfection aid reagent, following the manufacturer’s protocol. All the sequences used are listed in Supplementary Table 2.

**Transwell migration and invasion assay**

Transwell cell culture chambers with 8 μm pore size polycarbonate membrane (Corning) were employed to determine the migratory and invasive capabilities of pancreatic cancer cells in this study. Briefly, cells (6-10 ×10^4^/well) with varying treatments were seeded into the upper chamber in 200 μL of serum-free medium, while 800 μL of complete medium containing 10 % FBS was placed in the lower chamber to act as a chemoattractant. For the invasion assay, the membrane was first coated with Matrigel™ (Corning) diluted at a 1:15 ratio with medium. After an incubation period of 24 hours at 37 °C, the filters were fixed in 4% paraformaldehyde for 15 minutes and stained with crystal violet for 20 minutes. The cells on the upper side of the filter were gently wiped off using a cotton swab. Ultimately, the chambers were photographed under a microscope at ×200 magnification and the number of cells was counted using ImageJ software.

**Western blot**

Total cellular protein was extracted using RIPA lysate. Proteins were subjected to 10% or 12.5% SDS-PAGE gels, followed by transferring onto PVDF membranes (Millipore). The PVDF membranes then were blocked in 5% skimmed milk for 1 hour and incubated overnight at 4 °C with specific antibodies including FUBP1 (ZENBIO, R24364), c-MYC (Proteintech, 10828-1-AP), GAPDH (Zhong Shan-Golden Bridge, TA-08) or β-actin (Zhong Shan-Golden Bridge, TA-09). After extensive washing, the membranes were incubated with corresponding secondary antibodies (Zhong Shan-Golden Bridge, ZB-2301, ZB-2305) for 1.5 hours. Immunocomplexes were visualized using electrochemiluminescence reagent (Millipore).

**RNA-pulldown assay and RNA immunoprecipitation (RIP)**

Biotin-labeled tsRNA and antisense probes were synthesized by Sangon Biotech (Shanghai, China). A total of 1 × 10^7^ cells were washed with ice-cold phosphate-buffered saline (PBS) and cell lysates were prepared using standard lysis buffers. The lysates were then incubated with 200 nM of biotinylated RNA oligo probes against endogenous expressed tsRNA at room temperature for 2 hours. After washed out unbound proteins on beads, bound proteins were detected by western blot or LC-MS/MS.

For RIP assay, magnetic beads were utilized to capture either anti-FUBP1 (ZENBIO, R24364) or anti-IgG (CST, 2729S) antibody. Prewashed 3 × 10^7^ cells with ice-cold phosphate-buffered saline (PBS) were lysed using lysis buffer (20 mM Tris-HCl pH7.5, 150 mM NaCl, 1.5 mM MgCl_2_, 10% glycerol, 0.5% NP-40, 0.5% Triton X-100). The antibody coupled-beads were then incubated with total RNA lysate overnight with rotation at 4 °C. After washed out unbound proteins, co-captured RNAs were purified by phenol chloroform and further detected by RT-qPCR.

**Immunohistochemistry**

Immunohistochemistry (IHC) assay was carried out according to a protocol described previously. Briefly, slides were subjected to exfoliation and rehydration and antigen repair followed by FUBP1 antibody (ZENBIO) incubation at a dilution of 1:300 overnight at 4°C. Then, slides were incubated with goat anti-rabbit antibody (Jackson) at a dilution of 1:250 for 40 minutes at room temperature. Signalstain DAB substrate kit (Cell Signaling Technology) was used for colorimetric detection. The intensity of staining was classified into four-categories: 0, no staining; 1, weak; 2, moderate; and 3, strong. The proportion of staining was scored as follows: 0, 0%–5%; 1, 6%–25%; 2, 26%–50%; And 3, >50%. The results of intensity x proportion of each slide were calculated, and the product >3 was considered as high expression, and product ≤3 was considered as low immunostained signal.

**Protein stability analysis**

We investigated the effect of tsRNA on the stability of FUBP1 protein in pancreatic cancer cells using CHX and MG132 treatments and detection of ubiquitination levels. Specifically, pancreatic cancer cells with control and overexpression of tsRNA were treated with 100 μg/mL CHX at different time points (0, 2, 4, 6, 8, 10 hour). Protein samples were collected and western blot was used to detect the change of FUBP1 protein level. Pancreatic cancer cells with control and overexpression of tsRNA were treated with 10 μM of MG132 at different time points (0 or 4 hours for gain-of-function experiments, and 0 or 12 hours for loss-of-function experiments). Samples were collected and subjected to detect FUBP1 expression by western blot. To analyze the effect of tsRNA on FUBP1 protein stability, FUBP1 immunoprecipitation was conducted in pancreatic cancer cells overexpressing tsRNA and exogenous ubiquitin.

**Chromatin immunoprecipitation (ChIP) assay**

Immunofluorescence assay was carried out according to a protocol described previously. Briefly, cells were treated with the crosslinking reagent formaldehyde (Sigma) at a final concentration of 1% for 10 minutes at room temperature, followed by quenching with glycine (final concentration of 0.125 M) for 5 minutes. After washed twice with cold phosphate-buffered saline (PBS), cells were lysed with lysis buffer (50 mM Tris pH 8.0, 10 mM EDTA, 1% SDS) containing protease inhibitors. The lysate was sonicated for 25 cycles (5 seconds "on" and 5 seconds "off" at 10% power) to shear the chromatin to an average size of 200-500 bp. After centrifuged at 12,000 rpm for 10 minutes at 4°C, the supernatant was collected as the chromatin extract. The chromatin extract was then divided equally and incubated overnight with either anti-FUBP1 antibody (ZENBIO) or anti-IgG (CST) antibody at 4°C. The chromatin-bound antibodies were captured using Protein A/G magnetic beads (MCE) for 2 hours at 4°C. The beads were then washed with washing buffer (50 mM Tris pH 8.0, 500 mM NaCl, 1% Triton X-100) and eluted with elution buffer (0.1 M NaHCO3, 0.5% SDS) at 65°C for 30 minutes. The eluted chromatin was treated with proteinase K (Thermo) to reverse the crosslinks, and the DNA was purified using a PCR clean-up kit (TaKaRa). The purified DNA was amplified by quantitative PCR (qPCR) using the SYBR mix (Vazymen) on a real-time PCR machine (Bio-Rad). The qPCR was performed using primer pairs designed to amplify specific regions of the genome of interest (Supplementary Table 3). The data obtained from qPCR was analyzed using the ΔΔCt method, with the input chromatin as the control.

***In vivo* metastasis analysis**

We investigated the impact of tsRNA on tumor metastasis *in vivo* by conducting animal assays on 4-week-old BALB/c Nude mice. Random assignment to each group was performed, with 6 mice per group. CFPAC-1 cells were transfected with either antagomir or NC and then injected *via* the tail vein (1.5 × 10^6^ cells per mouse) into the nude mice. Imaging was performed and metastasis was recorded 4-weeks post-injection. As for rescue animal experiment, 4-week-old NCG mice were used, with 5 mice in each group. PANC-1 cells were transfected with either agomir or NC and then injected *via* the tail vein (1.5 × 10^6^ cells per mouse) into the nude mice. Imaging was performed and metastasis was recorded 20-days post-injection. These mice were procured from Gempharmatech Company (Chengdu, China) and housed at the Experimental Animal Center of West China Hospital of Sichuan University. Animal experiments were performed in accordance with the guidelines of the Experimental Animal Welfare Ethics Committee, West China Hospital of sichuan University (approval number: 20220505002).

**Statistical analysis**

The statistical analysis in this study was performed using GraphPad Prism 7.0 software. All data were presented as means ± SD or means ± SEM, and appropriate two-tailed unpaired or paired Student’s *t* tests were utilized for analysis. Survival curves were generated using the Kaplan-Meier method, and the log-rank test was used to determine any significant differences. Furthermore, the Cox proportional hazards regression model was utilized to identify predictive factors that independently affected the prognosis of pancreatic cancer. All statistical tests were two-sided, and statistical significance was set at *p* < 0.05.

**Supplementary Table 1 Primers used in this study.**

| Name | Sequences (5 ́-3 ́) |
| --- | --- |
| tiRNA-Val-CAC-2 RT | GTCGTATCCAGTGCAGGGTCCGAGGTATTCGCACTGGATACGACGAGGCG |
| tiRNA-Lys-CTT-3 RT | GTCGTATCCAGTGCAGGGTCCGAGGTATTCGCACTGGATACGACGGGTCT |
| tiRNA-Lys-TTT-3-M2 RT | GTCGTATCCAGTGCAGGGTCCGAGGTATTCGCACTGGATACGACAAGTCT |
| tiRNA-Lys-CTT-1-M2 RT | GTCGTATCCAGTGCAGGGTCCGAGGTATTCGCACTGGATACGACGAGTCC |
| tiRNA-Val-CAC-1-M3 RT | GTCGTATCCAGTGCAGGGTCCGAGGTATTCGCACTGGATACGACGAGGCG |
| tiRNA-Val-CAC-2 PCR R | GTCATCCTTGTAGTCGGATCCCGCCTCGTCGTATCCAGTGC |
| tiRNA-Val-CAC-2 PCR F | ATTTCCGGTGAATTCCTCGAGGCTTCTGTAGTGTAGTGGTTATCACGT |
| tiRNA-Lys-CTT-3 PCR R | GTCATCCTTGTAGTCGGATCCAGACCCGTCGTATCCAGTGCG |
| tiRNA-Lys-CTT-3 PCR F | ATTTCCGGTGAATTCCTCGAGGCCCGGCTAGCTCAGTCG |
| tiRNA-Lys-TTT-3-M2 PCR R | GTCATCCTTGTAGTCGGATCCAGACTTGTCGTATCCAGTGCGAA |
| tiRNA-Lys-TTT-3-M2 PCR F | ATTTCCGGTGAATTCCTCGAGGCCCGGATAGCTCAGTCGG |
| tiRNA-Lys-CTT-1-M2 PCR R | GTCATCCTTGTAGTCGGATCCGGACTCGTCGTATCCAGTGCG |
| tiRNA-Lys-CTT-1-M2 PCR F | ATTTCCGGTGAATTCCTCGAGGCCCGGCTAGCTCAGTCG |
| tiRNA-Val-CAC-1-M3 PCR R | GTCATCCTTGTAGTCGGATCCCGCCTCGTCGTATCCAGTGC |
| tiRNA-Val-CAC-1-M3 PCR F | ATTTCCGGTGAATTCCTCGAGGTTTCCGTAGTGTAGTGGTTATCACG |
| tiRNA-Val-CAC-2 qPCR F | TCGGCAGGCTTCTGTAGTGTAG |
| tiRNA-Lys-CTT-3 qPCR F | ATTGCCCGGCTAGCTCAGT |
| tiRNA-Lys-TTT-3-M2 qPCR F | TAGCCCGGATAGCTCAGTCG |
| tiRNA-Lys-CTT-1-M2 qPCR F | ATTGCCCGGCTAGCTCAGT |
| tiRNA-Val-CAC-1-M3 qPCR F | GCACGTTTCCGTAGTGTAGTGG |
| tsRNA qPCR R | CCGAGGTATTCGCACTGGAT |
| U6 qPCR F | AACGCTTCACGAATTTGCGT |
| U6 qPCR R | CTCGCTTCGGCAGCACA |
| FUBP1 qPCR F | AACAGGACCTCCAGACCGATGT |
| FUBP1 qPCR R | TCCAGTTGCCTTGACCTCTACC |
| c-MYC qPCR F | CCTGGTGCTCCATGAGGAGAC |
| c-MYC qPCR R | CAGACTCTGACCTTTTGCCAGG |
| GAPDH qPCR F | GTCTCCTCTGACTTCAACAGCG |
| GAPDH qPCR R | ACCACCCTGTTGCTGTAGCCAA |

**Supplementary Table 2 Mimic and inhibitor sequences of tiRNA-Val-CAC-2 used in this study.**

| Oligo name | Sequences (5 ́-3 ́) |
| --- | --- |
| Mimic NC | UUGUACUACACAAAAGUACUG |
| Mimic tiRNA-Val-CAC-2 | GCUUCUGUAGUGUAGUGGUUAUCACGUUCGCCUC |
| Inhibitor NC | CAGUACUUUUGUGUAGUACAA |
| Inhibitor tiRNA-Val-CAC-2 | GAGGCGAACGUGAUAACCACUACACUACAGAAGC |

**Supplementary Table 3 Primers used for ChIP in this study.**

| Name | Sequences (5 ́-3 ́) |
| --- | --- |
| c-MYC Site 1 F | AATGGTAGGCGCGCGTAGTT |
| c-MYC Site 1 R | CGAGCACTCTAGCTCTAGGATGTA |
| c-MYC Site 2 F | GGGACCAAGGATGAGAAGAATG |
| c-MYC Site 2 R | TTGTTTGCTCCCTGAAATGATC |
| c-MYC Site 3 F | AGGTGGTGGAGGGAGAG |
| c-MYC Site 3 R | TATATTCCCTCGGGATTTTTTATTTTGTG |

**Supplementary Table 4 Clinical characteristics of tissue specimens from primary and metastatic pancreatic cancer.**

| Group | ID | Gender | Age | Location site | Stage | CEA  (ng/mL) | CA199  （U/ml） |
| --- | --- | --- | --- | --- | --- | --- | --- |
| Metastatic tumor | M1 | Female | 57 | Liver | IV | ＞1000 | ＞1000 |
|  | M2 | Male | 54 | Liver | IV | 3.04 | 44.7 |
|  | M3 | Male | 63 | Liver | IV | 1.28 | ＞1000 |
| Primary tumor | P1 | Male | 57 | Pancreas | III | 3.52 | 30.4 |
|  | P2 | Male | 49 | Pancreas | III | 2.27 | 719 |
|  | P3 | Female | 54 | Pancreas | IB | 2.15 | 110 |

**Supplementary Table 5 The counts per million mapped reads of the selected five tsRNAs in 6 tumor samples by tsRNA sequencing**

| ID | Sequence | Length | Fold_Change | *p*_value | M1 | M2 | M3 | P1 | P2 | P3 |
| --- | --- | --- | --- | --- | --- | --- | --- | --- | --- | --- |
| tiRNA-Lys-TTT-3-M2 | GCCCGGATAGCTCAGTCGGTAGAGCATCAGACTT | 34 | 6.885 | 0.006 | 1007 | 2140 | 114.2 | 72.54 | 64.39 | 79.03 |
| tiRNA-Val-CAC-2 | GCTTCTGTAGTGTAGTGGTTATCACGTTCGCCTC | 34 | 5.357 | 5E-04 | 2209 | 3911 | 1724 | 394.6 | 305.8 | 111.8 |
| tiRNA-Lys-CTT-3 | GCCCGGCTAGCTCAGTCGGTAGAGCATGAGACCC | 34 | 4.846 | 0.002 | 129.7 | 202.5 | 91.92 | 21.76 | 14.08 | 8.947 |
| tiRNA-Lys-CTT-1-M2 | GCCCGGCTAGCTCAGTCGGTAGAGCATGGGACTC | 34 | 4.659 | 0.012 | 8301 | 18308 | 3538 | 1329 | 462.8 | 1051 |
| tiRNA-Val-CAC-1-M3 | GTTTCCGTAGTGTAGTGGTTATCACGTTCGCCTC | 34 | 2.781 | 0.039 | 624.9 | 1587 | 973.5 | 127.7 | 395.4 | 87.97 |

Fold_Change: 2^(log2FC); *p*_value, The *p*-value of the exact test by negative binomial distribution.

**Supplementary Table 6 Detailed binding sites of tiRNA-Val-CAC-2 to FUBP1 amino acids is also predicted by HDOCK database.**

| Receptor-ligand interface residue pair(s) | | | | | |
| --- | --- | --- | --- | --- | --- |
| 283A - 6A | 316A - 34A | 347A - 26A | 352A - 23A | 354A - 23A | 368A - 18A |
| 283A - 7A | 317A - 7A | 348A - 26A | 352A - 24A | 355A - 17A | 368A - 19A |
| 312A - 5A | 317A - 8A | 349A - 16A | 353A - 17A | 355A - 21A | 376A - 22A |
| 312A - 6A | 318A - 7A | 349A - 17A | 353A - 20A | 355A - 22A | 432A - 22A |
| 312A - 7A | 318A - 8A | 349A - 26A | 353A - 21A | 358A - 16A | 433A - 21A |
| 313A - 7A | 318A - 9A | 350A - 24A | 353A - 22A | 358A - 17A | 433A - 22A |
| 315A - 34A | 319A - 7A | 350A - 26A | 353A - 23A | 358A - 18A | 436A - 21A |
| 316A - 7A | 320A - 6A | 351A - 24A | 354A - 17A | 365A - 18A | 436A - 22A |
| 316A - 8A | 320A - 7A | 352A - 17A | 354A - 21A | 367A - 18A | 437A - 21A |
| 316A - 33A | 346A - 26A | 352A - 17A | 354A - 22A | 367A - 19A | 440A - 21A |
| 441A - 21A |  |  |  |  |  |


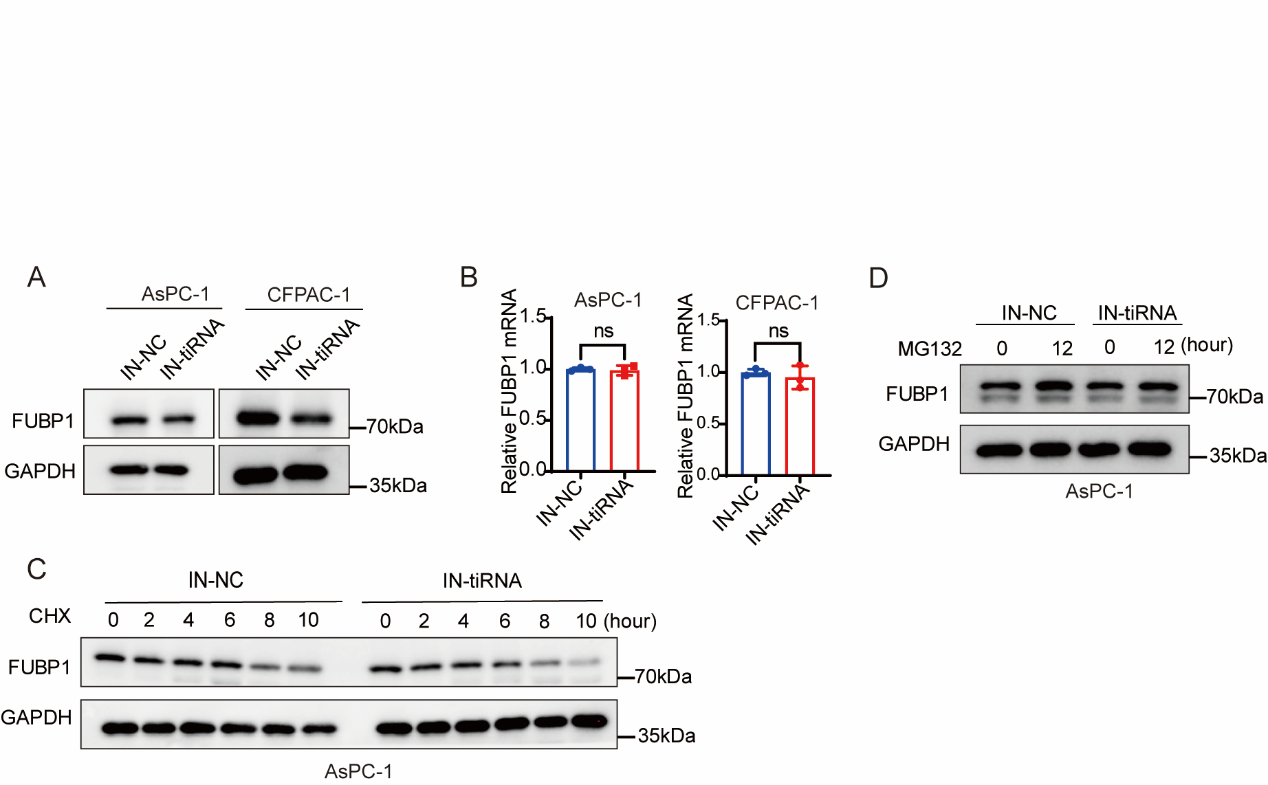


**Supplementary Fig.1 Knockdown tiRNA-Val-CAC-2 inhibits the stability of FUBP1 protein.** (A) FUBP1 expression was detected by western blot in tiRNA-Val-CAC-2 knockdown AsPC-1 and CFPAC-1 cells. (B) The mRNA level of FUBP1 was detected by RT-qPCR in cells same as panel A. (C) After the AsPC-1 cells were treated with 100 μg/mL Cycloheximide (CHX), the half-life of FUBP1 protein was determined by western blot. (D) The effect of tiRNA-Val-CAC-2 on the stability of FUBP1 protein was tested by western blot in AsPC-1 cells treated with 10 μM of MG132. IN-NC: inhibitor NC; in-tsRNA: inhibitor tiRNA-Val-CAC-2. ns, no significance


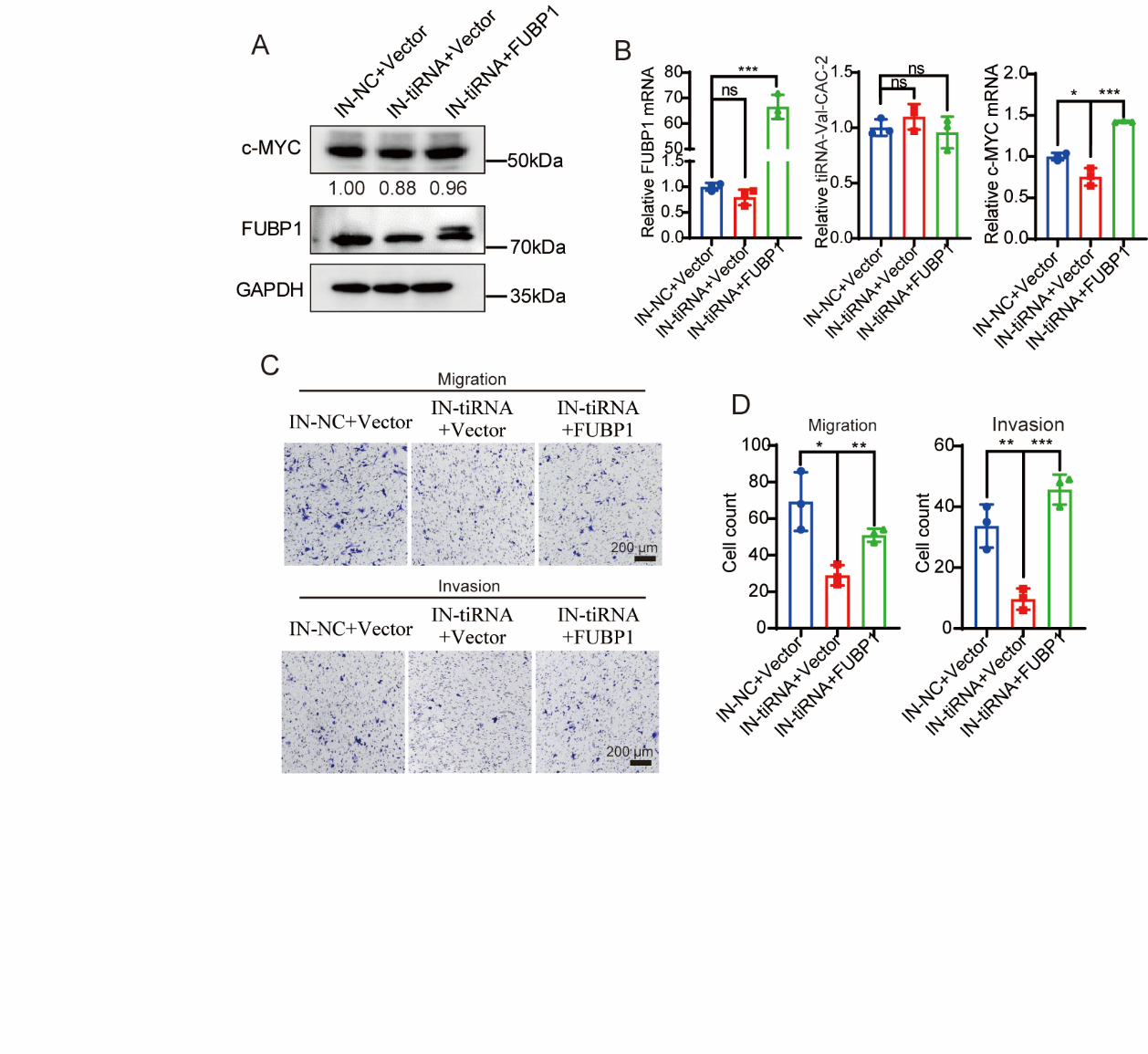


**Supplementary Fig.2** **Knockdown of tiRNA-Val-CAC-2 inhibits the metastasis by down-regulating *c-MYC* transcription through FUBP1.** (A-B) The protein (A) and mRNA (B) level of c-MYC was measured in indicated cells by western blot and RT-qPCR. (C-D) The migration and invasion abilities were measured in indicated cells by transwell assay. IN-NC: inhibitor NC; in-tsRNA: inhibitor tiRNA-Val-CAC-2. ns, no significance; * *p*<0.05, ***p*<0.01, ****p*<0.001.
